# Supplementary material for: Achieving the UNAIDS first ‘95’ in prenuptial HIV/AIDS testing among reproductive-aged Rwandese women: A multilevel analysis of 2019–20 population-based data
Source: PLOS Glob Public Health. 2023 Feb 15;3(2):e0001033. doi: 10.1371/journal.pgph.0001033 (PMC10021437; doi:10.1371/journal.pgph.0001033)
Supplement: Attachment — Submitted filename: Point-by-point response.docx [file pgph.0001033.s001.docx]

October 18, 2022

Manuscript ID: [PGPH-D-22-01238] - [EMID:b2c467cef3e1ae8b]

**Title:** “Achieving the UNAIDS first ‘95’ in prenuptial HIV/AIDS testing among reproductive-aged Rwandese women: a multilevel analysis of 2019-20 population-based data”

RE: Revised manuscript submission and response to reviewers’ comments

Dear Editor,

This letter is in reference to your email dated October 17, 2022 with reviewers’ comments. We are very pleased that the manuscript is potentially acceptable for publication in *Plos Global Public Health* once we have carried out the revisions.

We would like to thank the reviewers for these insightful and helpful comments and for giving us the chance to revise our manuscript. We believe the revised manuscript has been significantly improved and the reviewers’ comments have been addressed adequately. We think in its current form it will make a valuable contribution to the literature on this increasingly important topic. 

Please find for your kind consideration the followings:

1) A section-by-section response to the comments and suggestions of the reviewers (below).
2) The revised manuscript, provided as a marked-up copy and a clean copy.

We hope that these changes meet with your favourable consideration. Please do not hesitate to get in touch if you require any further information. 

Amadou Barrow
Corresponding Author 

Reviewer #1

I congratulate the authors for selecting a topic that has strong policy implications for achieving the UNAIDS' first ‘95’ target. The manuscript does not have page and line numbers, for which I will only focus on major comments instead of specific ones. My major comments are regarding the manuscript's methodological aspects.

**Response**: Thank you very much for the insightful comments. We have included line numbers to the revised version.

First, the prevalence of prenuptial HIV/AIDS testing was found to be high, i.e., 45.9% (95% CI: 44.8-47.1). In the situation where the binary outcome is common, i.e., the prevalence is greater than 10%, the odds ratio (OR) estimated from binary logistic regression overestimates or jeopardizes the true measure of association (for more information, please see Ranganathan et al. 2015 and Tamhane et al. 2016). In such cases, a prevalence ratio (PR) instead of OR is the appropriate option. Authors should consider using Poisson regression models with robust variance, reporting Adjusted Prevalence Ratios (APR) and 95% Confidence Intervals (CIs). The authors may also consider using log-binomial or other appropriate methods for calculating the adjusted PR.

**Response**: We agree that the prevalence of prenuptial HIV/AIDS testing was high. However, we adjusted for sampling weights, clustering and stratification. This is to ensure that the study design does not affect the estimates (true measure of association). Poisson regression is not used for modeling “binary outcome”. Poisson is used for modeling “count outcome”. Therefore, poisson regression as recommended is not suitable to be used for modeling our “binary outcome”.

Moreover, when specifying log-binomial model in multilevel analysis, random intercept model using meglm for the log-binomial regression:

“*meglm depvar indvar1 indvar2 indvar3,…,indvar_n || community: || household:, family(binomial) link(log) eform*”,

We consistently got error message.

But when this is done without considering the hierarchical nature of the data, the results were well displayed from Stata. Since our initial analysis confirmed significant variance by levels of data, we proceeded with the multilevel multivariable logistic regression which is widely used in literature. We hope this is accepted provided our approach is technically sound.

Second, the authors correctly stated that given the hierarchical nature of the DHS data, a multilevel model would be an appropriate analytical approach. Multilevel analysis is also a suitable approach to take into account the social contexts as well as the individual respondents. My major concern is about the authors’ choice of levels for the analyses. To me, it was not clear how the levels were selected and this needs to be described and supported. Have the authors used any theoretical framework or solely relied on the DHS sampling frame used for Rwanda? For example, if the authors had used a conceptual framework of health services utilization, viz., Aday (1974), Andersen and Newman (1973), Andersen (1995), then following levels would seem suitable:
1. Individuals/household (n=13,000)
2. Clusters/EAs (n=500)
3. Districts (n=30)
4. Provinces (n=5)

**Response**: Thank you very much for the insightful comments. Our selection was strictly based on statistical significance for a level variance. For example, the household-level variance was not statistically significant. Hence, we did not need to create the “household-level”, but include the variables to the individual-level. The lead author of this paper is a proficient and trained biostatistician. He ensure there was no analytical lacking left to compromise the quality of the results. Even if you have pre-determined levels from literature, they must also be tested to establish their suitability. The choice of selection has been provided in the paper as below:

Statistical Analysis (Line 206):

*“The mixed-effects of the factors associated with prenuptial HIV/AIDS testing were estimated using an adjusted multilevel binary logit model. For binary response reporting prenuptial HIV/AIDS testing, we developed a two-level model for individual women (at level 1) and communities/enumeration areas (at level 2). These were the levels with significant variances. We created four models. The null or empty model without any factor, was created to examine community-level variance. This is used to understand how much community factors could explain the observed variations. When the variance is statistically significant, the use of a multilevel model is justified. If the community-level variance was not significant, the single-level logit model is recommended. The second model contains individual compositional factors only, while the third model has the cluster-level factors only. The fourth model adjusted for individual compositional and cluster/community-level factors”*.

Another example is that population-based survey data usually have hierarchical nature that requires the conduct of multilevel regression analysis. However, researchers and biostatisticians would use the "variance value" from the null model to justify the use of multilevel statistical framework, because if the “cluster variance” is not significant in the empty model, it advised to use the “usual” regression model. These are the rules we followed throughout this study.

Lastly, it is not clear how the authors developed the socioeconomic status (SES) index. Have they used rural location and unemployment in developing the SES index? How it differs from the standard asset score needs to be described. Including "unemployment" in the standard list for developing asset score may be problematic, as the assets indicate the household's long-term socioeconomic status, and unemployment may indicate an individual-level recent phenomenon. Also, keeping location (rural/urban) a separate indicator would give you more information on the effect of place of residence on HIV testing services use.


References:
Aday LA, Andersen RM. A framework for the study of access to medical care. Health Serv Res 1974;9(3):208-220.

Andersen RM, Newman JF. Societal and individual determinants of medical care utilization in the United States. Milbank Memorial Fund Quarterly– Health and Society 1973;51(1):95-124.

Andersen RM. 1995. Revisiting the Behavioral Model and access to medical care: does it matter? Journal of Health and Social Behavior 36: 1–10.

Ranganathan P, Aggarwal R, Pramesh CS. Common pitfalls in statistical analysis: Odds versus risk. Perspect Clin Res. 2015;6(4):222. DOI: 10.4103/2229-3485.167092.

Tamhane AR, Westfall AO, Burkholder GA, Cutter GR. Prevalence odds ratio versus prevalence ratio: choice comes with consequences. Stat Med. 2016;35(30):5730–5. DOI: 10.1002/sim.7059.

**Response**: Thank you very much. On how we developed the socioeconomic status (SES) index, we used a secondary dataset in this study which contains variables on residential status, household wealth quintiles, education and employment status. Using complex Stata command, respondents who dwell in rural residence, poorest, lack formal education and unemployed were computed using principal component analysis to generate socioeconomic variable classified as low, medium/moderate and high (Line 171-173). This approach has also been used in previous studies:

gen rural = V025 ==2

gen pov = V190 == 1

gen ilt = V106 == 0

gen ntw = V717 == 0

gen commune = V021

bysort commune: gen total = _N

egen ilx = total(il), by(commune)

egen uex = total(ntw), by(commune)

egen povertyx = total(pov), by(commune)

bysort commune: gen com_poverty = (povertyx / total)*100

bysort commune: gen com_illit = (ilx / total)*100

bysort commune: gen com_uemp = (uex / total)*100

global ses "rural com_poverty com_illit com_uemp"

qui factor $ses, pcf

qui predict com_zses

xtile com_zsesc3 = com_zses, nq(3)

tab com_zsesc3

recode com_zsesc3 (1= 1 "low") (2 = 2 "medium") (3 = 3 "high"), gen(SES)

tab SES

Reviewer #2

Comments are uploaded in the file attached. I have the following comments:
1. The article has no line numbers

**Response**: We have included the line numbers as requested. Thank you.

2. Abstract, methods: include the location of the data if one is interested in re-analyzing the data

**Response**: The location of the data is already provided in the methods: “*This study included a total of 14,634 reproductive-aged* ***Rwandese*** *women using 2019-20* ***Rwanda*** *Demographic and Health Survey (RDHS)*”. Thank you.

3. Abstract, results: include the odds ratio, 95% confidence intervals, and p-values of all the factors found

**Response**: Considering that we have 300 words limit in the abstract, we have revised as much as possible. Thank you very much.

4. Abstract, conclusion: Were the recommendations derived from this study? What was their odds ratio and p-values to show that they worked?

**Response**: We have revised the “conclusion” in line with the findings from the study. Thank you very much for the insightful comments.

5. Main results: provide p-values for the variables in the models, besides having them in the table

**Response**: Thank you for your suggestion. The inference to be reached by p-values, that is, the statistical significance when p<0.05 is the same with the 95% confidence intervals (when the interval does not contain the value “1”) already provided in Table 3 for instance. As much as we have checked, there is no multilevel regression results that included p-values alongside the confidence intervals in result tables. We would appeal to keep our results without p-values based on literature. We hope you will agree with us on this.

6. References: Some of them are duplicated eg 4 and 6, 23 and 25.

**Response**: References 4 and 6 have been corrected. However, references 23 and 25 are different. While the reference 23 is from 2014-15 survey, reference 25 is from 2019-20 survey respectively. Thank you.

7. Some of the tables are more than a page long, try to shorten them.

**Response**: Thank you for your recommendation. We followed the guidelines of the journal. However, we believe that, if accepted, the production team would format the tables in line with the journals requirements.
