# Supplementary material for: Achieving the UNAIDS first ‘95’ in prenuptial HIV/AIDS testing among reproductive-aged Rwandese women: A multilevel analysis of 2019–20 population-based data
Source: PLOS Glob Public Health. 2023 Feb 15;3(2):e0001033. doi: 10.1371/journal.pgph.0001033 (PMC10021437; doi:10.1371/journal.pgph.0001033)
Supplement: Attachment — Submitted filename: Point-by-point response.docx [file pgph.0001033.s002.docx]

December 20, 2022

Manuscript ID: [PGPH-D-22-01238R1] - [EMID:721133b41adedd44]

**Title:** “Achieving the UNAIDS first ‘95’ in prenuptial HIV/AIDS testing among reproductive-aged Rwandese women: a multilevel analysis of 2019-20 population-based data”

RE: Revised manuscript submission and response to reviewers’ comments

Dear Editor,

This letter is in reference to your email dated December 5, 2022 with reviewers’ comments. We are very pleased that the manuscript is potentially acceptable for publication in *PLOS Global Public Health* once we have carried out the revisions.

We would like to thank the reviewers for these insightful and helpful comments and for giving us the chance to revise our manuscript. We believe the revised manuscript has been significantly improved and the reviewers’ comments have been addressed adequately. We think in its current form it will make a valuable contribution to the literature on this increasingly important topic. 

Please find for your kind consideration the followings:

1) A section-by-section response to the comments and suggestions of the reviewers (below).
2) The revised manuscript, provided as a marked-up copy and a clean copy.

We hope that these changes meet with your favourable consideration. Please do not hesitate to get in touch if you require any further information. 

Amadou Barrow
Corresponding Author 

Reviewer #1: I thank the authors for incorporating feedback and detailed responses. I am highlighting below the issues that require the authors’ attention:

First, the authors’ statements that Poisson regression is for count data and not suitable for modeling our binary outcome are not correct. Poisson models can be used for the binary outcome and, in fact, there are options for Poisson models (viz., using robust error variances or quasi-Poisson models) that provide correct estimates and are a better alternative for the analysis of cross-sectional studies with binary outcomes than logistic regression. Given the high prevalence of prenuptial HIV/AIDS testing, using the odds ratio instead of the prevalence ratio (or interpreting the odds ratio as if it were a prevalence ratio) is not a technically sound approach not only in terms of the possible overestimation, but also because confounding may not be appropriately controlled. There are published articles on how logit models are widely misused in public health/epidemiological studies. Apart from the references I already stated in my original feedback, the authors may review the highly cited article by Barros & Hirakata (2003) from <http://www.biomedcentral.com/1471-2288/3/21> in this regard.

**Response**: Thank you very much for the insightful comments. We know from literature that Poisson regression is primarily for count data, while logistic regression for modeling binary outcome variable.

1. Gardner, W., Mulvey, E.P., and Shaw, E.C (1995). “Regression Analyses of Counts and Rates: Poisson, Overdispersed Poisson, and Negative Binomial Models”, Psychological Bulletin, 118, 392-404.
2. Long, J.S. (1997). Regression Models for Categorical and Limited Dependent Variables, Chapter 8. Thousand Oaks, CA: Sage Publications.
3. https://stats.oarc.ucla.edu/stata/seminars/regression-models-with-count-data/

In addition, for modeling the high prevalence of prenuptial HIV/AIDS testing using logistic regression, we followed similar approach from previous studies:

1. Antenatal service utilization of 62.8%: https://bmcpregnancychildbirth.biomedcentral.com/counter/pdf/10.1186/s12884-020-03236-9.pdf?pdf=button%20sticky
2. Optimum antenatal care visit of 56%: https://bmcpregnancychildbirth.biomedcentral.com/counter/pdf/10.1186/s12884-021-04362-8.pdf?pdf=button%20sticky
3. Skilled birth attendance of 67.18%: https://ijponline.biomedcentral.com/counter/pdf/10.1186/s13052-020-00943-z.pdf?pdf=button%20sticky
4. Postnatal care for both mother and newborn of 85.6%: https://bmcpublichealth.biomedcentral.com/counter/pdf/10.1186/s12889-022-12494-5.pdf?pdf=button%20sticky
5. Postnatal care of 57.5%: <https://bmcpregnancychildbirth.biomedcentral.com/counter/pdf/10.1186/s12884-018-2138-x.pdf?pdf=button%20sticky>

We would appeal to the reviewer that our paper should not be rejected only for this reason provided we have followed previous studies approach. However, we have noted your suggestions and hope to explore widely in our future papers.

Second, you are getting an error message while using log-binomial models in multilevel analysis because, while using meglm, a log link is not allowed with binomial or Bernoulli family due to convergence issues. The authors may review the help file for meglm where the allowable combinations for links and families are provided in a table.

**Response**: Thank you very much for your insightful comment. As it was essential for us to conduct multi-level analysis, whereas the meglm does not run with the log link in log-binomial models, we think we could keep the mixed-effect estimates.

Third, the authors responded that their approach to developing the SES index has also been used in previous studies. Given the unusual approach used for the SES index, I’d recommend the authors to provide a few references to support this claim. In the manuscript, the authors cited references # 29 and 30. These studies used PCA for developing household wealth indices, but neither used rurality, lack of formal education, unemployment, and poorest quintile (which, in turn, was derived using PCA itself) to create an index using PCA. Also, information on respondent’s education and employment multiple times under different variables (viz., educational attainment, working status, SES, Enlightenment). Have the authors tested for multicollinearity in their regression model?

**Response**: Thank you very much. In the statistical analysis sub-section of the methods, we reported the collinearity testing approach: “*A variance inflation factor analysis was used to examine the presence of multicollinearity [Ref. 34]. In the absence of multicollinearity, significant variables from the Chi-square test were retained in the logit model*”

In addition, we present herewith, previous studies that used: rural residence, poorest, lack formal education and unemployment to compute socioeconomic disadvantage/deprivation variable:

1. https://reproductive-health-journal.biomedcentral.com/counter/pdf/10.1186/s12978-018-0658-5.pdf?pdf=button%20sticky
2. https://jepha.springeropen.com/counter/pdf/10.1186/s42506-020-00041-2.pdf
3. https://www.thelancet.com/action/showPdf?pii=S2468-2667%2818%2930111-7
4. https://jech.bmj.com/content/jech/57/9/699.full.pdf
5. https://journals.plos.org/plosone/article/file?id=10.1371/journal.pone.0241416&type=printable
6. https://www.thelancet.com/action/showPdf?pii=S2468-2667%2822%2900036-6
7. https://www.ncbi.nlm.nih.gov/pmc/articles/PMC6284478/pdf/khvi-14-10-1504524.pdf
8. <https://jepha.springeropen.com/counter/pdf/10.1186/s42506-020-00064-9.pdf>

Finally, the manuscript would be benefitted from proofreading and copyediting as there are issues with punctuation and subject-verb agreement throughout the manuscript.

**Response**: Thank you very much for the insightful comments. We have revised the manuscript for typos.
